# Autophagy-dependent ferroptosis in infectious disease

**DOI:** 10.2478/jtim-2023-0099

**Published:** 2023-12-20

**Authors:** Jiarou Li, Hongliang Wang

**Affiliations:** Department of Critical Care Medicine, The Second Affiliated Hospital of Harbin Medical University, 246 Xuefu Road, Nangang District, Harbin 150086, Heilongjiang Province, China; Future Medical Laboratory, The Second Affiliated Hospital of Harbin Medical University, 246 Xuefu Road, Nangang District, Harbin 150086, Heilongjiang Province, China

**Keywords:** autophagy, ferroptosis, infectious disease, sepsis, selective autophagy

## Abstract

Autophagy is the initial defense response of the host against pathogens. Autophagy can be either non-selective or selective. It selectively targets the degradation of autophagic substrates through the sorting and transportation of autophagic receptor proteins. However, excessive autophagy activity will trigger cell death especially ferroptosis, which was characterized by the accumulation of lipid peroxide and free iron. Several certain types of selective autophagy degrade antioxidant systems and ferritin. Here, we summarized the latest researches of autophagy in infection and discuss the regulatory mechanisms and signaling pathways of autophagy-dependent ferroptosis.

## Introduction

Lysosomes have long been regarded as the “garbage-disposal system” for cells because of the more than 60 acid hydrolases.^[1]^ Nevertheless, the function is more than breaking down cell components, foreign molecules, or pathogens^[2]^. In recent years, researchers have gained a better understanding of lysosomes by combining genomics, transcriptomics, proteomics, bioinformatics, and other methods. Lysosomes are now regarded as regulators of cell homeostasis that mediate signal transduction, metabolic adaptation, cell proliferation, the quality control of proteins and organelles, and even programmed cell death.^[3, 4, 5]^ The process by which selected cellular components are transported to and broken down within lysosomes is called Macroautophagy. Macroautophagy is a physiological process that contributes to the high conservation of intracellular homeostasis. In addition, autophagy also occurs selectively. The soluble or membrane-binding receptors recognize cargo and mediate the formation of autophagosomes. The selective sequestration into autophagosomes depends on the interaction between the cargo-binding receptor and Autophagy-related protein 8 (ATG8) family proteins anchored on the membrane forming autophagosome. Excessive or dysfunctional autophagy and cellular killing are highly correlated.^[6]^ In particular, the term “autophagy-dependent cell death” was recommended by the Nomenclature Committee on Cell Death to describe a form of ferroptosis-regulated cell death (RCD) that mechanistically depends on the autophagic machinery or components.^[7]^ In this narrative review, we will discuss the latest evidence for the significant role of autophagy-mediated ferroptosis in infectious diseases.

## Infection and inflammation initiate autophagy

At present, the diagnosis, treatment, and prevention of infectious diseases worldwide are still facing severe challenges.^[8]^ Sepsis is the number one healthcare cost and cause of death in hospitals.^[9]^ Shock and multi-organ failure from Sepsis may lead to adverse outcomes.^[10]^

Infection-induced autophagy is initiated by the binding of pathogen-associated molecular patterns within the microbial structure to pattern recognition receptors, such as toll-like receptors.^[11, 12]^ This activates different intracellular events and leads to increased autophagic activity by promoting the conversion of microtubule-associated protein1 light chain 3-I to -II (LC3 to LC3-II) in turn.^[13]^ As such, binding of lipopolysaccharides of Gram-negative pathogens onto toll–like receptor 4 (TLR4) activates autophagy via the p38/ Mitogen-Activated Protein Kinase (p38/MAPK) signaling axis whereas binding of lipoteichoic acid to TLR2 induces autophagy by the MAPK1/ERK2-MAPK3/ERK1 pathway. Different TLR ligands exhibit variable autophagyinducing abilities. Single-stranded RNA binding to TLR7 is the most efficient inducer.^[11, 14]^

### Defense function of moderate autophagy

There are at least four general pathways that may be used for autophagy-protein-dependent targeting of bacteria to the lysosome. These include the autophagy-proteinfacilitated fusion of pathogen-containing phagosomes with lysosomes, the envelopment of bacteria-containing phagosomes or endosomes by autophagosomal membranes, the fusion of bacteria-containing phagosomes or endosomes with autophagosomes, or the xenophagic capture of bacteria that have escaped inside the cytoplasm.^[15, 16]^ LC3 / ATG8 is cleaved by ATG4 at the carboxyl end to produce cytoplasmic LC3-1. LC3-1 is coupled with phosphatidylethanolamine (PE) through ATG7 and 3 (corresponding to E1 and E2-like enzymes respectively) to produce lipidized LC3, also known as LC3-l1, which can be attached to the membrane of autophagosome.^[16, 17]^ It is the structural protein of autophagosomes.

**Figure 1:**
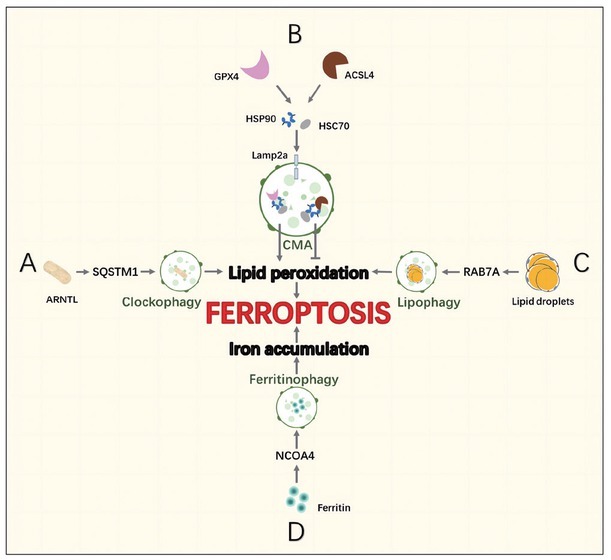
Known pathways involving the autophagy machinery by which viruses and bacteria was targeted to the lysosome. Cathepsin B (CTSB) can directly cleaves the lysosomal calcium channel mucolipin 1 / transient receptor potential mucolipin 1 (MCOLN1/TRPML1). The inactive transcription factor EB(TFEB) is unable to induce transcription of lysosomal and autophagyrelated genes.

Cellular cargo is commonly targeted to autophagosomes by interactions between a molecular tag (such as polyubiquitin), adaptor proteins such as p62 or neighbor of BRCA1 Gene 1 (NBR1) (which recognize these tags and contain an LC3-interacting region (LIR) characterized by a WXXL or WXXI motif), and LC3. These adaptor molecules can mediate the selective recognition and degradation of aggregates by autophagy by binding ubiquitin chains on aggregates and key protein LC3 on the autophagy membrane (Figure 1).

Cysteine cathepsin is an important driving element of lysosomal work. They also play an indispensable role in autophagy, antigen presentation, cell stress signaling, metabolism, and lysosome-dependent cell death. Here, cathepsin B (CTSB) regulates the bioavailability of lysosomes and autophagosomes and plays an essential role in maintaining a precise balance between protein synthesis and degradation in lysosomes.^[18]^ Under steady conditions, CTSB cleaves calcium channel mucolipin 1 / transient receptor potential mucolipin 1 (MCOLN1/TRPML1) in the lysosomal membrane, maintains the low level of transcription factor EB (TFEB), and reduces the expression of lysosomal and autophagy-related genes. This regulation stabilizes the number of lysosomes and autophagosomes in cells. As a negative feedback regulator of lysosomal biogenesis and autophagy, CTSB is a potential target of various bacterial infections. Mice and macrophages lacking cathepsin B activity showed obvious resistance to the cytosolic pathogen Francisella novicida CTSB-knockout animals produced fewer pro-inflammatory cytokines and chemokines in the liver.^[19]^ Moreover, F. novicida exploited the activity of CTSB, resulting in permissiveness to bacterial replication and increasing susceptibility to infection.

To summarise, CTSB provides an apical signal controlling the expression of lysosomal and autophagy-related proteins as a way of maintaining the population of lysosomes and autophagosomes within a cell (Figure 1).^[20]^

The master modulator Transcription factor EB (TFEB) promotes both autophagy and lysosomal biogenesis via upregulating relevant genes. Increasing shreds of evidence have linked TFEB and the Subsequent Lysosome biogenesis with pathogenetic mechanisms and control of sepsis. TFEB-dependent xenophagy and autophagylysosome pathway (ALP) is critical for antimicrobial defense and therapeutic applications of sepsis. TFEB-activating agents, such as trehalose,^[21]^ bedaquiline,^[22]^ and acacetin,^[23]^ enhanced bactericidal activity when the body is infected by intracellular pathogens (*e.g.*, Mycobacterium tuberculosis and Salmonella typhimurium). In summary, TFEB has been recognized as the pharmacological target of their potential in treating sepsis.^[24]^

However, if TFEB is directly used as a drug to treat sepsis, its safety and effectiveness are still unconvincing. Both the status of autophagy and the activity of TFEB are altered during the progression of sepsis. Therefore, time-dependent changes in the expression and activity of TFEB in sepsis are needed to be evaluated.

### Excess autophagy trigger ferroptotic cell death

As the genes involved in NF-κB signaling, autophagy and lysosomal protein degradation were synchronously enriched in the model of septic.^[25, 26]^ It is beginning to be recognized that Altered autophagy function results in maladaptive inflammation and more severe disease.^[27]^ Autophagy has its physiological roles and too much or too little autophagy can both be harmful. Deficient autophagy may lead to acute infection and tissue aging while excessive autophagy could result in cytokine storm and even cell death. ^[15, 28]^

Previous studies have demonstrated that autophagy is involved in the regulation of endothelial permeability during inflammation.^[29–31]^Moreover, the production, processing, and secretion of cytokines, such as IL-1 also need the participation of autophagy.^[25, 32]^ Based on this theoretical basis, many people hypothesize that autophagy may represent a potential therapeutic strategy for preventing excessive inflammation during sepsis. Experimental results indicate that inhibition of autophagy at either initiation stage by 3-MA or autophagosome and lysosome fusion stage by CQ after E. coli infection have several beneficial effects.^[33]^ It has hinted that inhibition of autophagy (at either initiation stage or autophagosome and lysosome fusion stage) after E. coli infection have several beneficial effects. preventing the over-production of pro-inflammatory cytokines and the development of endothelial hyperpermeability in mice. Autophagy inhibitors did not weaken the ability of immune cells to clear bacteria.

The relationship between autophagy and inflammatory factors was firstly explored in yeast.^[34]^ Autophagy can enhance the activation of caspase-1 through a type of ATG5-dependent non-classical pathway, promoting the activation of the inflammasome and thereby increasing the synthesis of IL-1β and IL-18. In the meantime, any pro-inflammatory factors cannot be decomposed through the ER into autophagy due to the lack of signal peptides. Instead, Autophagy promotes the migration of these proinflammatory factors to the cytoplasm and aggravates the inflammatory damage of tissues.

In a recent study lipopolysaccharide (LPS)-induced ARDS both cellular and animal model demonstrated that IL-1β in secretory autophagosomes participate (SAP) in the pathogenesis of ARDS by mediating the inflammatory response and lung injury and this mechanism is associated with ras-related protein Rab-8A (RAB8a). Accordingly, SAPs may serve as a novel marker, and RAB8a may be a potential therapeutic target in ARDS. ^[35]^

## Ferroptosis is a common phenomenon in infectious diseases

Iron is an essential nutrient for both humans and pathogenic microbes. After infection, it causes competition for metals between bacterial pathogens and hosts^[36, 37]^. Bacteria extract Fe^3+^ from tissues, body fluids, cells, and proteins by secreting siderophores, heme acquisition systems, transferrin or lactoferrin receptors, and ferric or ferrous iron transporters. In contrast, hosts resist bacteria by isolating Fe^3+^ in binding proteins and ferritin.^[38]^

So far, many different kinds of bacterial infections have been confirmed to be associated with the activation of ferroptosis. For example, the Gram-negative bacterium aeruginosa can oxidize AA-PE to 15-hydroperoxy-AA-PE (15-HOO-AA-PE), and lipid peroxidation triggers ferroptosis in human bronchial epithelial cells. This chronic airway inflammation eventually leads to cystic fibrosis.^[39–41]^ The ability of Pseudomonas aeruginosa isolates in patients with persistent lower respiratory tract infections to induce ferroptosis depends on the level and enzymatic activity of pLoxA.^[39]^ This process can be blocked by baicalein (an ALOX inhibitor) and ferrostatin- 1 (a ferroptosis inhibitor). With the new sight into the pathogenic mechanism of PA, it has been demonstrated that PA degrades the glutathione peroxidase-4 (GPX4) defense by activating the lysosomal chaperone-mediated autophagy (CMA).^[40]^

Mycobacterium tuberculosis (Mtb) caused tuberculosis (TB) is the leading cause of death by a single infectious agent. Macrophage necrosis after infection is the main cause of bacterial spread and disseminated disease.^[42]^ Mtbinduced macrophage necrosis is associated with reduced levels of glutathione and GPX4, along with increased free iron, lipid peroxidation, and mitochondrial superoxide, all of which are characteristic marks of ferroptosis.^[43]^ Moreover, necrosis in infected macrophage cultures was well suppressed by ferrostatin-1, a well-characterized ferroptosis inhibitor.

Recently more and more studies reported that ferroptosis is involved in the pathological development in the mouse model of polymicrobial sepsis induced by cecal ligation and puncture. Significant and specific changes of ferroptosis related indexes were detected in Multiple Organ Dysfunction caused by sepsis, such as heart,^[44, 45]^ liver,^[46]^ lung,^[39, 47]^ and kidney.^[48, 49]^ The lack of GPX4, FSP1, DHOD, and other defense system pathways is the main pathogenesis of ferroptosis.^[50, 51]^

Interestingly, ferroptosis has also been proven to be an important factor in viral infectious diseases. Many kinds of viruses will interfere with iron uptake, directly use iron transporters to enter cells or damage the antioxidant response system, resulting in ferroptosis.^[52]^ There is growing evidence showing that ferroptosis may be a key component of the process leading to Multiorgan Damage pulmonary and extrapulmonary manifestations in COVID-19.^[52, 53]^ In 2020, Jacobs et al. Published an autopsy report of a 48-year-old male patient who died of COVID-19 despite extracorporeal life support,^[54]^ RRT and maximum drug treatment. The myocardial and proximal tubules of the kidney tissue were examined for markers of ferroptosis. Are indicators representing the degree of lipid peroxidation during ferroptosis, and their immunohistochemical stain are positive. These results highlight ferroptosis is probable to contribute to COVID-19 cardiac damage and multiple organ failure detrimentally.

Current research shows that there are three proposed mechanisms of Ferroptosis after sars-cov-2 infection: Dysregulation of Iron Metabolism, GSH-GPX4 Axis, and ROS over Generation. Abnormal expression of various proteins related to iron metabolism can be found in patients with COVID- 19. The infection ^[55, 56]^ may cause the degradation of ferritin through “ferritinophagy”^[53, 55]^ and triggers the increase in a labile iron pool that increases the production of lipid peroxidation and ROS via the Fenton reaction and, eventually, promotes ferroptosis. In addition to this way, sars-cov-2 can also overproduce ROS by combining the TOM70 at the surface of the mitochondria membrane and triggering NOX. Targeting intracellular defense response, SARS-CoV-2 significantly suppressed mRNA expression of GPX4,^[56]^ DNA synthesis-related thioredoxin reductase, and endoplasmic reticulum-resident selenoproteins.

In clinic, biomarkers related to ferroptosis show a good function of predicting the prognosis of patients with sepsis. Ferritin is an indicator of total iron load, hence it showed a better association in long-term survival analysis.^[57]^ High transferin saturation (TSAT) presented a substantial predictive value for both short and long-term survival in septic patients, whereas low transferrin levels were only significantly associated with short-term mortality.^[51, 58]^

**Figure 2:**
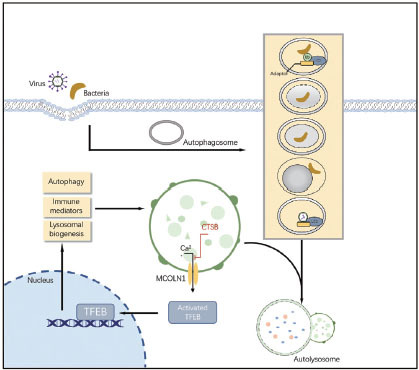
The role of different types of selective autophagy in ferroptosis. (A) RAB7A-mediated lipophagy, and (C) SQSTM1-mediated clockophagy promote lipid peroxidation in ferroptosis (B) Chaperone-mediated autophagy (CMA) blocks lipid peroxidation by degrading ACSL4 and promotes lipid peroxidation by degrading GPX4 in ferroptosis (D) NCOA4-mediated ferritinophagy promotes iron accumulation.

## Molecular mechanism of autophagy-dependent ferroptosis

In addition to inflammatory response, autophagy activation caused by infection also plays a multifaceted regulator of cell death. The recently discovered function of autophagy, especially selective types of autophagy (*e.g.*, ferritinophagy, lipophagy, clockophagy, and chaperone-mediated autophagy), in driving cells towards ferroptosis motivated us to explore the functional interactions between metabolism, lysosome, and cell death (Figure 2).

### NCOA4-mediated ferritinophagy

Ferritinophagy is the process of autophagic degradation of the iron-storage protein ferritin, which is critical for the regulation of cellular iron levels.^[59]^ A quantitative proteomic assay has unveiled a role for nuclear receptor coactivator 4 (NCOA4) as a selective cargo receptor for ferritinophagy, which interacts with an arginine residue in the C terminal domain of FTH1, accounting for selective sequestration and degradation of ferritin and elevated Fe^2+^ bioavailability in the cytosol. This delivery via macroautophagy enables cells to use stored iron. Meanwhile, the increase of free iron level provides the possibility of ferroptosis.^[60, 61]^

The NCOA4-ferritin axis modulates intracellular iron homeostasis in accordance with cellular iron availability.^[62, 63]^ In the LPS induced sepsis myocardial injury model, we can see that the protein changes of NCOA4 and ferritin are closely related in a time-dependent manner.^[62]^ LPS promotes the expression of NCOA4 and interaction between ferritin, which eventually leads to ferroptosis. NCOA4-ferritinophagy also participated in the progress of periodontitis, an inflammatory disease mostly caused by Porphyromonas gingivalis. This gram-negative anaerobe triggered the activation of c-Jun N-terminal kinase (JNK) and p38 mitogen-activated protein kinase signaling pathway. Ferritinophagy, which comes from NCOA4 transcription, aggravated production of the ROS and inflammatory responses in PDLFS.^[64]^

These findings suggest NCOA4 mediates iron homeostasis and plays an important role in the pathogenesis of infectious diseases.

### RAB7A related Lipophagy

The lipid droplet proteome fluctuates with changes in cellular metabolic status, thus influencing the composition of neutral lipids in organelles.^[65, 66]^ Lipophagy, the autophagic digestion of lipid droplets, can release free fatty acids, which then serve as a fuel for mitochondrial beta-type oxidation. The process by which intracellular lipid droplets are selectively transported by autophagosomes for lysosomal decomposition. Lipophagy provides another potential pathway for regulating cellular lipid levels and therefore, the propensity to ferroptosis.^[67]^ RAB7A, a cargo receptor for autophagic LD, degradation promotes RSL3-induced ferroptotic cell death in hepatocytes. Lipid droplet accumulation is increased at the early stage but decreased at the late stage of ferroptosis in mouse or human hepatocytes.^[66]^ Importantly, either genetically enhancing TPD52-dependent lipid storage or blocking ATG5-and RAB7A-dependent lipid degradation prevents RSL3-induced lipid peroxidation and subsequent ferroptosis in vitro and in vivo.^[67]^ These studies support an antioxidant role in stable lipid droplets in cell death.

### Chaperone-mediated autophagy

GPX4 is an irreplaceable anti-oxidant enzyme in cells.^[68]^ It can reduce lipid peroxidation and inhibit the process of ferroptosis. The decrease of GPX4 quantity or activity is the clear mechanism of ferroptosis.^[69]^ With the constant deepening of research on ferroptosis, it is found that the degradation of GPX4 protein is a pivotal event.^[63, 70, 71]^

Chaperone-mediated autophagy (CMA) is a type of selective autophagy that uses molecular chaperones to deliver certain cytosolic proteins to lysosomes for degradation based on the recognition of KFERQ-like motif within the sequence of a protein. ^[70, 72]^ Heat shock protein family A (Hsp70) member 8 (HSPA8/HSC70), and HSP90 are major molecular chaperones responsible for the recognition and later degradation.^[63, 70, 73]^ Moreover, HSP90 can increase the stability of lysosomal-associated membrane protein 2A (LAMP2A), a CMA receptor, to assist GPX4 degradation. These findings establish a model of the interrelationship between CMA and ferroptosis. However, the structural basis of GPX4 degradation needs to be explored.

Degradation of GPX4 protein by CMA seems to be a common response to various ferroptosis activators.^[74–76]^ Moreover, CMA-induced ferroptosis can be observed in inflammatory models caused by different injury factors, such as aseptic inflammation caused by pancreatitis,^[77]^ radiation,^[78]^ and ischemia-reperfusion,^[79]^ and infection we mentioned above^[39, 40]^. Under damage conditions, the host GPX4 defense was degraded by activating the lysosomal chaperone-mediated autophagy (CMA), and the autophagy inhibitor Baf-A1 significantly increased the level of GPX4 and alleviated injury.

However, the role of CMA in ferroptosis may not be as simple as expected. A recent article shows that the activation of CMA may also specifically degrade ACSL4, which contains six possible KFERQ-like motifs. A fatty acid-activating enzyme, long-chain fatty acyl-CoA synthetase 4 (ACSL4) participates in fatty acid metabolism and then contributes to the production of lipid peroxidation ^[80]^. In early diabetic retinopathy, Glia maturation factor-β induces ferroptosis by impairing chaperone-mediated autophagic degradation of ACSL4, resulting in the accumulation of lipid peroxidation.

### SQSTM1-dependent clockophagy

CMA contributes to the rhythmic removal of clock machinery proteins and to the circadian remodelling of a subset of the cellular proteome. It has been show that “clockophagy,” the selective degradation of the core circadian clock protein -Aryl hydrocarbon receptor nuclear translocator like protein (ARNTL) by autophagy, is critical for ferroptosis.^[81, 82]^ ARNTL inhibits ferroptosis by repressing the Egl nine homolog 2 (Egln2), thus activating the prosurvival transcription factor hypoxia inducible factor-1 (HIF1A). Measures to prevent ARNTL degradation or inhibit Egln2 activation can destroy the stability of HIF1A and promote the ferroptosis of tumor cells.^[82, 83]^ Sequestosome 1(SQSTM1) is a multifunctional cargo receptor implicated in the autophagic degradation of ubiquitinated substrates, including proteins and organelles. Mass spectrometric analysis showed that SQSTM1 was the interactor of ARNTL under normal conditions. In RSL3-induced ferroptosis, immunoprecipitation analysis showed increased SQSTM1-ARNTL interaction.^[81]^

## Conclusions and perspectives

Cytokine storm and organ damage caused by infectious diseases are life-threatening problems that have plagued people for a long time. With an in-depth understanding of the pathogenic mechanism of infection, it is found that autophagy-mediated ferroptosis dominate in cell injury and death. Excessive activation of autophagy can lead to degradation of intracellular defense or beneficial components. Here, we discuss recent progress in molecular mechanisms of autophagy-mediated ferroptosis, providing insights into all the networks of relationships between lysosome, infection, and ferroptosis.
